# Topoisomerase II-binding protein 1 promotes the progression of prostate cancer via ATR-CHK1 signaling pathway

**DOI:** 10.18632/aging.103260

**Published:** 2020-05-27

**Authors:** Kaiwen Li, Shirong Peng, Zean Li, Yiming Lai, Qiong Wang, Yiran Tao, Wanhua Wu, Qianghua Zhou, Ze Gao, Junxiu Chen, Hui Li, Wenli Cai, Zhenghui Guo, Hai Huang

**Affiliations:** 1 Department of Urology, Sun Yat-Sen Memorial Hospital, Sun Yat-Sen University, Guangzhou, China; 2 Guangdong Provincial Key Laboratory of Malignant Tumor Epigenetics and Gene Regulation, Sun Yat-Sen Memorial Hospital, Sun Yat-Sen University, Guangzhou, China; 3 Department of Biochemistry and Molecular Genetics, University of Virginia School of Medicine, Charlottesville, VA 22903, USA; 4 Department of Radiology, Massachusetts General Hospital, Harvard Medical School, Boston, MA 02114, USA

**Keywords:** TopBP1, DDR, prostate cancer, ATR-CHK1

## Abstract

DNA damage response (DDR) plays an important role in the progression of cancers, including prostate cancer (PCa). Topoisomerase II-binding protein 1 (TopBP1) is an essential promotor of ATR-mediated DDR. Herein, we investigated the association between TopBP1 and PCa and determined its effect on the progression of PCa. The expression and clinical features of TopBP1 were analyzed using large-scale cohort of tissue microarray analyses and The Cancer Genome Atlas database, which indicated that TopBP1 was positively correlated with high Gleason Score, advanced clinical and pathological stages, the metastasis status. Multivariate analysis revealed that the upregulation of TopBP1 was an independent predictor for a worse biochemical recurrence-free survival (BCR-free survival). Furthermore, we discovered that downregulation of TopBP1 significantly suppressed the growth and migration ability of PCa lines by loss-of-function assays *in vitro.* Further mechanistic investigations clarified that TopBP1 promoted proliferation and migration by activating ATR-Chk1 signaling pathway.

## INTRODUCTION

Prostate cancer (PCa) is one of the most common cancers among men worldwide, accounting for 15% of all cancers diagnosed [1]. In 2019, approximately 20% of new expected cancer cases and 10% of the cancer death among American males are PCa [2]. Furthermore, the incidence and mortality of PCa in developing countries also appears increasing [3]. Traditionally, various combinations of Gleason score (GS), stage, lymph node status, and prostate-specific antigen (PSA) are used to classify the risk and predict prognosis after local treatment of PCa [4]. However, PSA testing might lead to over diagnosis frequently, which accounts for 23% to 42% of all screen-detected cancers and brings more aggressive treatments [5]. Besides, many prostate cancers are indolent, and do not need aggressive surveillance after radical prostatectomy (RP). Thereafter, it is essential to better understand the underlying mechanisms of progression and identify more accurate predictors or therapeutic targets for PCa.

The development of PCa are closely related to DDR which includes detection of DNA damage, accumulation of DNA repair factors and physical repair of the lesion [6, 7]. DNA strand breakage, one of the main types of DNA damage, enables Topoisomerase II-binding protein 1 (TopBP1) to induce ATR-Chk1 mediated checkpoint activation and DNA repair [8]. TopBP1 was first identified as a factor interacting with C-terminal region of DNA topoisomerase IIβ [9]. TopBP1 is a ‘scaffold’ protein that makes numerous protein–protein interactions through their nine BRCT domains [10]. TopBP1 can enhance ATR kinase activity through interaction with ATR-interacting protein (ATRIP), thus initiating cell arrest and checkpoint activation [10]. *In vitro*, silencing expression of TopBP1 in PCa cells increases the sub-G1 cell population, which indicates the association between TopBP1 and PCa progression [11]. To date, the clinical significance of TopBP1 in PCa has not been fully evaluated yet. Our study showed the role of TopBP1 in PCa and its correlation with clinicopathological characteristics and the prognosis by analyzing the data from TCGA and immunohistochemistry of Tissue Microarray (TMA). And we found that TopBP1 enhanced proliferation and induced migration of PCa by activating ATR-Chk1 signaling pathway.

## RESULTS

### Association of TopBP1 expression with the clinicopathological characteristics of the patients with PCa

We first investigated whether the expression of TopBP1 is associated with clinical characteristics of PCa by using the publicly available TCGA database (Table 1). As shown in Table 1, increased TopBP1 expression in PCa patients was correlated with a higher GS (*p<*0.001), advanced pathological stages (p=0.004), and the presence of lymph node and distant metastasis (*p=*0.002 and *p=*0.033, respectively).

**Table 1:** The association of TopBP1 expression with clinicopathological characteristics in PCa patients.

	**TCGA**			**TMA**			
	**Case**	**$\bar{x}\pm s$**	**P**	**Case**	**Low, n (%)**	**High, n (%)**	**P**
**Tissue**							
Cancer	498	332.28±227.26		71	27 (38.0)	44 (62.0)	0.002*
Benign	NA	NA		7	7 (100.0)	0 (0)	
**Age**							
<60	201	558.58±194.24	0.243	24	9 (37.5)	15(62.5)	0.964
≥60	296	579.86±202.21		46	17 (37.0)	48(63.0)	
**Gleason score**							
<7	44	496.80±131.78	<0.001**	NA	NA	NA	
=7	247	526.56±174.41		NA	NA	NA	
>7	206	640.75±199.10		NA	NA	NA	
**Clinical stage**							
T1-T2	NA	NA		43	24 (55.8)	19 (44.2)	<0.001**
T3-T4	NA	NA		26	2 (7.7)	24 (92.3)	
**Pathological stage**							
T1-T2	351	541.25±175.28	0.004**	45	23 (51.1)	22 (48.9)	0.002*
T3-T4	55	659.74±283.07		24	3 (12.5)	21 (87.5)	
**Lymph node metastasis**							
N0	344	566.64±180.74	0.002**	57	26 (45.6)	31 (54.4)	0.003**
N1	80	639.99±234.06		12	0 (0)	12 (100)	
**Distant metastasis**							
M0	455	570.08±199.12	0.033*	60	26 (43.3)	34 (56.7)	0.012*
M1	3	820.57±538.28		8	0 (0)	8 (100)	

NA=data not available; TCGA=The Cancer Genome Atlas datase; TMA= tissue microarray.

· P<0.05, **P<0.01.

In order to validate the results of TCGA database, we detected TopBP1 protein expression in TMA. In the TMA, the expression profiles of TopBP1 in the 71 PCa and 7 normal tissues were examined. Similar to the TCGA database, Table 1 showed that TopBP1 expression was higher in PCa patients with advanced clinical and pathological stage (*p<*0.001 and *p=*0.002, respectively), and the presence of lymph node and distant metastasis (*p=*0.003 and *p=*0.012, respectively; Table 1).

### TopBP1 expression is upregulated in PCa tissues

**Figure 1:**
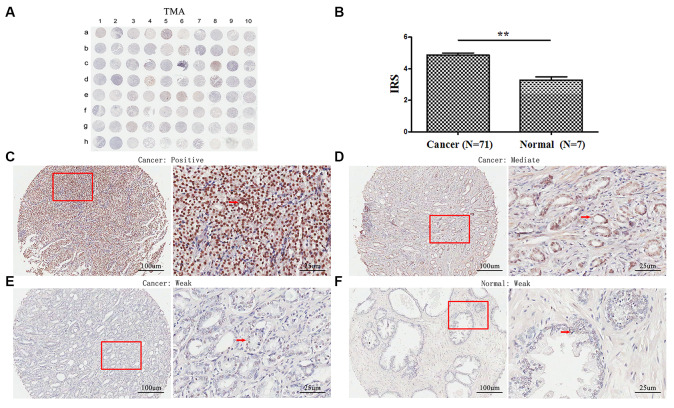
**Immunohistochemical staining for TopBP1 expression in prostate cancer and paracancerous tissues of our TMA sample.** (**A**) A full view of the immunohistochemistry staining for TopBP1 in TMA. (**B**) The immunoreactivity scores (IRS) of TopBP1 in prostate cancer (n=71) and in paracancerous tissues (n=7) Data were presented as Mean ± SEM. ^*^p = 0.002. (**C**–**E**) The immunohistochemistry staining indicated that TopBP1 immunostainings mainly occurred in the cytoplasm of PCa and the intensity of TopBP1 immunostainings was positive (**C**), intermediate (**D**), and weak (**E**). (**F**) Weak staining of TopBP1 in paracancerous tissues.

TMA analyses revealed that TopBP1 expressed mainly in the cytoplasm of most PCa tissues. However, weak staining was observed in the paracancerous tissues (Figure 1A, 1C–1F). The expression of TopBP1 in the PCa tissues was significantly higher than that of paracancerous tissues (IRS: 4.86±1.14 versus 3.29±0.49, respectively; *p*=0.002) (Figure 1B). Furthermore, in PCa tissues, 62.0 % of patients had high TopBP1 expression while 38.0 % low TopBP1 expression, which was significantly different from the distribution in paracancerous tissues (*p*=0.002, Table 1).

### Prognostic implications of TopBP1 expression in PCa

**Figure 2:**
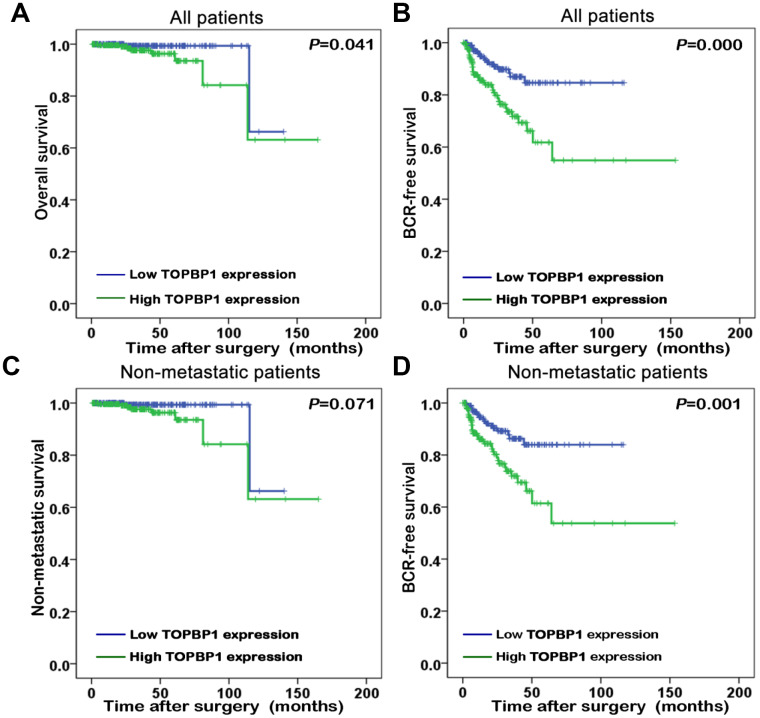
Kaplan-Meier survival curves of (**A**) overall survival and (**B**) biochemical recurrence (BCR)-free survival for TopBP1 expression in all patients with prostate cancer (PCa). (**C**) Non-metastatic survival and (**D**) BCR-free survival for TopBP1 expression in non-metastatic PCa patients.

The association of TopBP1 expression with the survival of PCa patients in TCGA database was evaluated by Kaplan-Meier plots. The median TopBP1 expression was used as the cut-off value to separate the PCa patients into high and low TopBP1 expression groups. Figure 2 illustrated patients with high TopBP1 expression had a worse overall and BCR-free survival compared with those with low TopBP1 expression in all patients (*p=*0.041 and *p<*0.001, respectively). While concerning non-metastatic patients at their primary diagnosis, only BCR-free survival was observed as significantly worse in high versus low TopBP1 expression group (*p*=0.001).

Univariate analysis implicated that BCR-free survival was significantly better in low versus high TopBP1 expression PCa patients (hazard ratio [HR]: 2.768; 95% confidence interval [CI]: 1.591-4.815; *p*<0.001) (Table 2). Furthermore, multivariate analysis demonstrated that upregulation of TopBP1 (HR: 2.130, 95% CI: 1.148-3.954; *p*=0.017), a higher GS (HR: 2.173, 95% CI: 1.207-3.909; *p*<0.01) and an advanced pathological stage (HR: 2.463; 95% CI: 1.341-4.522; *P*=0.004) were independent predictors for a worse BCR free-survival (Table 2).

**Table 2:** Univariate and multivariate analyses BCR-free survival in PCa patients.

**Variables**	**BCR-free survival**	
	**HR (95% CI)**	***p***
**Univariate analysis**		
Age (≥60 vs. <60)	1.319(0.773-2.250)	0.301
Gleason Score (<7 vs.=7 vs.>7)	3.175(1.881-5.360)	<0.001^**^
Pathological stage (T1-T2 vs. T3-T4)	3.950(2.226-7.008)	<0.001^**^
Lymph node stage (N0 vs. N1)	1.879(1.049-3.365)	0.034^*^
Distant metastasis (M0 vs. M1)	3.536(0.488-25.641)	0.212
TopBP1 expression (low vs. high)	2.768(1.591-4.815)	<0.001^**^
**Multivariate analysis**		
Gleason score (<7 vs.=7 vs.>7)	2.173(1.207-3.909)	0.010^**^
Pathological stage (T1-T2 vs. T3-T4)	2.463(1.341-4.522)	0.004^**^
TopBP1 expression (low vs. high)	2.130(1.148-3.954)	0.017^*^

HR= hazard ratio; CI= confidence interval; TopBP1= Topoisomerase II-binding protein 1; ^*^ *p*<0.05, ^**^p<0.01.

### TopBP1 promotes the proliferation of prostate cancer cells *in vitro*

**Figure 3:**
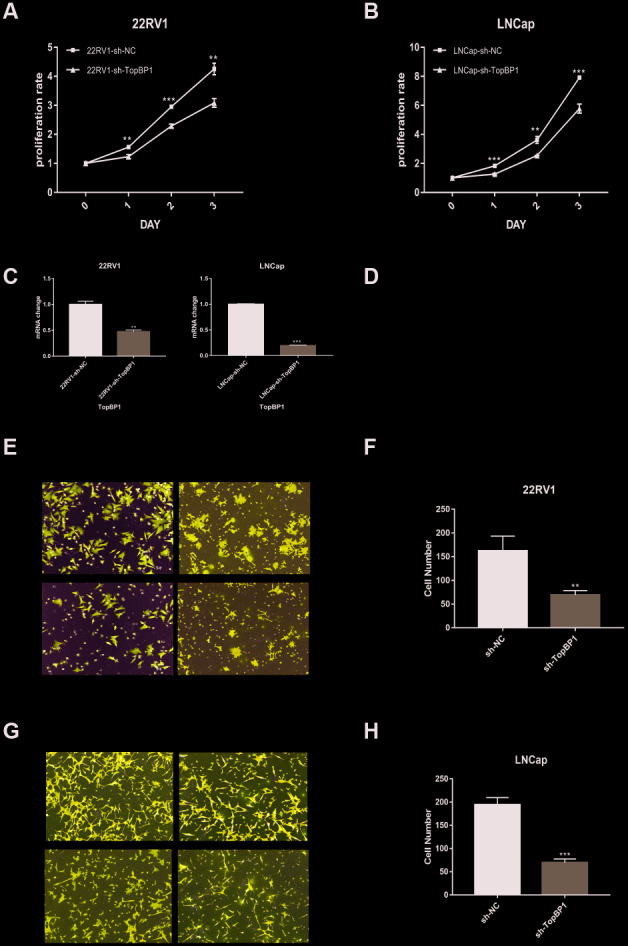
Down-regulation of TopBP1 significantly suppressed the proliferation of both 22RV1 (**A**) and LNCaP (**B**, **C**) qRT-PCR was performed to detect alteration of the TopBP1 expression. (**D**) Western blotting was performed to detect alteration of TopBP1. (**E**–**H**) Down-regulation of TopBP1 suppresses the migration of prostate cancer. (**E**, **G**) Represented images of two separated experiments in each cell line are showed. The data presented are mean ± SD for three independent experiments. **P<0.01 compared with NC, ***P<0.001 compared with NC.

To investigate the effect of TopBP1 on the proliferation abilities of prostate cancer cells, we knocked down the expression of TopBP1 in LNCaP and 22RV1 cells. By CCK8 assays, we found knocking down the expression of TopBP1could significantly inhibited the proliferation of both LNCaP and 22RV1 cells (Figure 3A–3D).

### Down-regulation of TopBP1 suppresses the migration of prostate cancer

As we mentioned above, TopBP1 expression was higher in PCa patients with the presence of metastasis. We performed transwell assays to evaluate migration ability in PCa cells. Knocking down the expression of TopBP1 significantly reduced migration ability of 22RV1 (Figure 3E, 3F), and LNCaP (Figure 3G, 3H).

### TopBP1 promotes the proliferation of prostate cancer cells *in vitro* by suppressing apoptosis through ATR-CHK1 signaling

**Figure 4:**
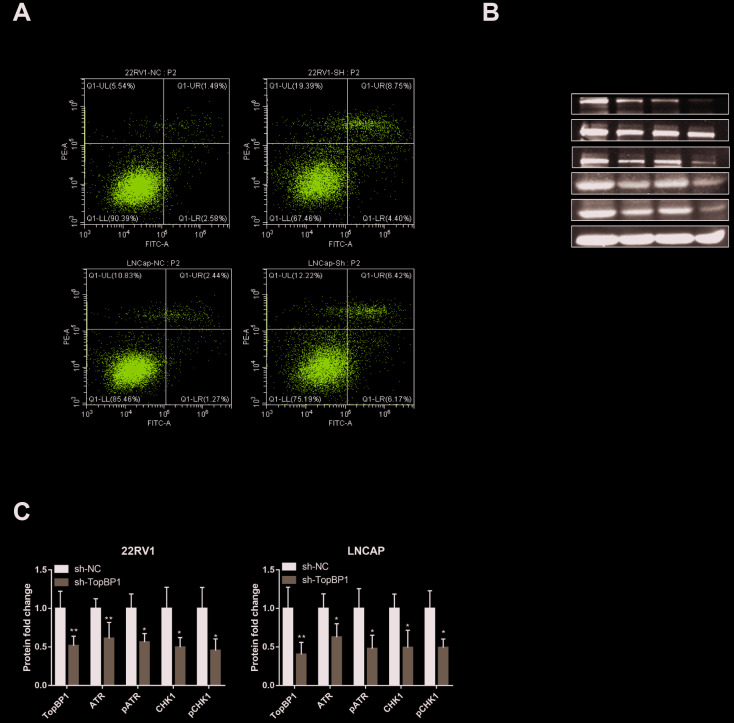
**Down-regulation of TopBP1 induced cell apoptosis.** (**A**) The expressions of ATR and Chk1 were decreased, followed by the decreased phosphoralation of ATR and Chk1. (**B**, **C**) The data presented are mean ± SD for at least three independent experiments. *P<0.05 compared with NC, **P<0.01 compared with NC.

In order to further explore the mechanism of TopBP1 in promoting the proliferation of PCa cells, we applied cell apoptosis assays. We found that knocking down TopBP1 increased apoptosis of both 22RV1 and LNCaP cells (Figure 4A). We then collected proteins of PCa cells and applied western blotting to detect ATR and Chk1. We found the expressions of ATR and Chk1, as well as phosphoyalation forms of ATR and Chk1, were decreased in both 22RV1 and LNCaP cells (Figure 4B, 4C). These results indicated that TopBP1 prevented PCa cells from apoptosis through ATR-Chk1 signaling.

## DISCUSSION

For this study, TopBP1 is hypothesized to have an important role in progression of PCa. By analyzing the TCGA dataset, higher expression of TopBP1 in PCa was significantly related to higher GS, advanced clinical stage, and higher rates of lymph node and distant metastasis. These results were validated by TMA analyses. Besides, TMA data also revealed that the expression of TopBP1 was higher in PCa than paracancerous tissue. Moreover, Kaplan-Meier analysis indicated that higher TopBP1 expression exhibited a worse overall survival and BCR-free survival in all patients. Cox regression analysis showed that TopBP1 can be an independent prognostic factor for BCR-free survival. Further study showed that TopBP1 may promote PCa through activating ATR-CHK1 signaling.

DDR refers to cellular mechanisms that prevent DNA damage accumulation and maintain genomic integrity, which plays a central role in survival of cells [6]. This response program has two coordinated functions: (i) to prevent or arrest duplication and partitioning of damaged DNA into daughter cells and (ii) to repair the damaged DNA and maintain the integrity of genome [12]. At the beginning of DDR, cell-cycle arrest will be activated, and then cells will attempt DNA repair. When DNA damage is promptly and properly repaired, cells will resume normal proliferation. However, if DNA damage is too severe for impossible fix, cells may undergo programmed cell death (apoptosis) to remove damaged cells, or become senescent (a naturally irreversible cell-cycle arrest) [13]. ATM/Chk2 pathway and ATR/CHk1 pathway is known to be DDR regulator in many cancers including prostate cancer [14–16]. NKX3.1, one of the prostate cancer suppressor, can interact with ATM leading to activate ATM, enhance the DDR and thus contribute to DNA integrity in prostate epithelial cells [17]. Meanwhile, Chk1 knockdown increases the sensitivity to radiotherapy by increasing DNA damage of PCa stem cells [18]. These data indicate that impaired DDR may initial prostate carcinogenesis while maintaining genomic integrity prevents the earlier stages of the prostate cancer through DDR activation [6]. Besides, it was reported that DDR defects increased the sensitivity to treatment with DNA-damaging agents [19].

TopBP1 acts an important role during activation of DDR and replication checkpoints [10]. TopBP1 can be recruited to the damage lesion and interact with the extreme C-terminal region of the 9-1-1 checkpoint clamp by a phospho-dependent mode [10, 20]. After that, TopBP1 can directly stimulate the kinase activity of ATR by the ATR and ATRIP interaction, leading to Chk1 phosphorylation and initiating checkpoint process [21, 22]. Recently, Karanika et al. reported that knockdown of androgen receptor (AR) or CDC6 reduces Chk1 S317 phosphorylation and induces apoptosis and cell death of PCa [23]. Besides, AR or CDC6 knockdown can also synergize with AZD7762, a Chk1/2 inhibitor, results in lower expression of TopBP1 and greater apoptotic effect in PCa cells [23], which indicates the promoting activity of TopBP1 in PCa. By using both clinicopathological data of TCGA and TMA, the present study revealed that TopBP1 expression had positive correlation with Gleason score, clinical stage, distant and lymph node metastasis. These results had high consistency with the *in vitro* study [23]. Moreover, TopBP1 is also thought to be an important regulator of DNA replication. TopBP1 could enhance CDC45 chromatin loading at DNA replication origins and activate replicative helicase which promote DNA replication initiation [24, 25]. Zhenkun Lou et al. reported that acetylation of TopBP1 in S phase was apparently higher than that in G1 phase, and acetylation of TopBP1 promote DNA replication by enhancing the TopBP1-Treslin interaction, CDC45 loading, and cell-cycle progression [26]. It may be possible that TopBP1 promotes PCa by preventing DNA damage and promoting DNA replication. However, we conducted flow cytometry for cell-cycle analyses and found no significant difference in the syntheses phase between TopBP1 shRNA and control cells. More importantly, depletion of TopBP1 did result in less DNA repair and increased cell apoptosis and DNA damage [23, 27]. Taken together, the main function of TopBP1 in PCa is preventing DNA damage instead of promoting DNA replication.

Alterations in expression of TopBP1 have been reported to be related to other cancers. Particularly, TopBP1 overexpression is found in 46 of 79 primary breast cancer tissues analyzed and is associated with high tumor grade and shorter patient survival time. The downstream effects of the overexpression are suggested to directly perturbing p53 function [28]. Seol et al. reported that the expression levels of TopBP1 and phosphorylated Chk1 were higher in radio-resistant when compared to radiosensitive lung cancer cell lines. They also observed that increased expression of TopBP1 had been highly correlated with more brain metastasis and reduced progression-free survival [29]. This study also indicated that higher TopBP1 expression had a reduced overall survival and BCR-free survival in all patients, while a reduced BCR-free survival in non- metastatic patients. All these data suggest that TopBP1 may be a good parameter for prediction of PCa prognosis.

Some limitations are still needed to be concerned. Firstly, in TMA analysis, sample size was small and the distribution of PCa and normal tissue was uneven, which might reduce its validation power. Secondly, some other factors may have effects on the prognosis of PCa patients, although Cox proportional hazard regression analyses had lower the possible confounding influence by the considerate variables.

## CONCLUSIONS

Our findings indicate that higher expression of TopBP1 in PCa is correlated with advanced cancer status and poor prognosis. Inherent in our findings is the implication that TopBP1 is a predictor for PCa prognosis and it may prevent prostate cancer from the accumulation of DNA damages via ATR-Chk1 signaling pathway.

## MATERIALS AND METHODS

### Patients and tissue samples

Tissue microarray (TMA, n=78) including 71 prostate cancer and 7 normal prostate tissues were obtained from Xi’an Alenabio Co, LTD (Cat No: PR803c). Tissues from patients with neoadjuvant chemotherapy or radiotherapy were excluded from the study. The Cancer Genome Atlas (TCGA) database including 498 patients with prostate cancer was collected for investigating the mRNA expression of TopBP1 level and performing the survival analysis.

### Immunohistochemistry staining analysis

TMA specimens were fixed in 10% neutral buffered formalin. Tissues were subsequently embedded in paraffin and at 4μm slide deparaffinized with xylene and rehydrated for peroxidase immunohistochemistry staining employing DAKO EnVision System (Dako Diagnostics, Switzerland). Following the proteolytic digestion and peroxidase blocking, tissue slides were incubated with the primary antibody against TopBP1 (rabbit polyclonal antibody, ab2402, Abcam Co. Ltd., UK) at a dilution of 1: 100, overnight at 4°C. Following washed with PBS, peroxidase labeled polymer and substrate-chromogen were used. Negative controls were carried out by omitting the primary antibody in each run.

### Immunostaining score evaluation

Two independent experienced pathologists, who were blinded to the detail information of the patients scored the intensity of immunostaining separately. Any discrepancies between them were resolved by re-evaluating the staining to achieve a consensus. According to the antibody specification sheet, cytoplasmic staining was considered as positive signals. In five representative fields at a 400-fold, the number of positively stained cells was counted and the percentage of positive cells was evaluated. TopBP1 expression was scored in a semi-quantitative method based on the intensity [negative (0 point), weak (1 point), moderate (2 points), and strong (3points)]and percentage [<5% (0 point), 6-25% (1 point), 26-50% (2 points), 51-75% (3 points), and >75% (4 points)]. A final immunoreactivity score (IRS) for each tissue was obtained by plusing the intensity level and the percentage of staining [30]. IRS more than 4 was grouped into high expression of TopBP1.

### Cell lines and cell culture

LNCaP and 22RV1 were purchased from the American Type Culture Collection (ATCC, Manassas, VA, USA). The cells were cultured in RPMI-1640medium (Gibco, Grand Island, NY, USA) with 10% fetal bovineserum (FBS), supplemented with 1% penicillin and streptomycin (Invitrogen, Carlsbad, CA, USA). Both cell lines were cultured in a humidifed incubator at 37°C with an atmosphere of 5% CO^2^.

### RNA isolation and Q-PCR

Trizol reagent (TaKaRa Biotechnology, Dalian, China) was used for extracting total RNA from cells and total RNA was reverse transcribed with a PrimerScript RT-PCR kit (Takara Biotechnology, Dalian, China) [31]. The Taqman TM RT-PCR system (Applied Biosystems, Carlsbad, USA) was used to conduct quantitative reverse transcriptase-polymerase chain reaction (qRT-PCR) according to the manufacturer’s protocol. The primers used are follows: TopBP1 forward: 5’- TTCAGCAACTCACAGTTAAGCA-3’ and reverse: 5’ GGCACACTCATACTTCTGACC-3’. PCR was performed in the presence of SYBR green (Sigma-Aldrich, St. Louis, USA) using 15 ng of total RNA with an ABI PRISM 7700 Sequence Detection System (Applied Biosystems).

### Transient transfection

RNA interference (siRNA) oligonucleotides targeting TopBP1 (5’-GCTAGAGTGTTTCAGTAAA-3’) and negative control siRNAs (5’-UUCUCCGAACGUGUCACGUTT -3’) were purchased from GenePharma (Shanghai, China).siRNA transfections were performed using 75 nM siRNA with 3 μl/ml Lipofectamine RNAimax (Life Technologies, Waltham, MA, USA) and incubated for 48 h for RNA isolation and 72 h for protein collection.

### Cell proliferation assay

CCK8 assay was used to determine the cell proliferation rate according to the manufacturer's protocol. Briefly, 3×10^3^ cells/well were seeded into 96-well plates (Corning, New York, NY, USA) containing 200 μl of RPMI-1640 medium containing 10% FBS and 1% penicillin streptomycin at 37°C and with 5% CO^2^ and were grown at 37°C for 24, 48 and 72 h. Subsequently, 20 μL CCK8 solution was added to each well and incubated in the dark for 2 h and optical densities (ODs) at 450 nm (OD450) were determined using a microplate reader (Multiskan MK3; Thermo Scientifc, Shanghai, China).

### Cell migration assay

Migration assay was performed by filling the bottom well of the cell culture insert (353097, Corning, USA) with RPMI 1640 medium containing 10% FBS. The insert wells were covered with polyethylene terephthalate (PET) membranes with 8-μm pores. Then 50,000 cells with 200ul serum-free RPMI 1640 were added to each top culture insert and was incubated for 24 h at 37°C for migration. At last, membranes were stained using crystal violet and migrating cells were counted through Olympus IX71 inverted microscope (Olympus, Japan).

### Apoptosis assay

The Annexin V/FITC apoptosis detection kit from BD was using to evaluate Apoptosis. Firstly, cells were seeded into 6-well plates and harvested by twice centrifugation at 1,000 rpm (5 min each spin). Then cells were washed twice (3 min each wash) in 1X PBS. Cells were resuspended in 100μl binding buffer containing 5 μl of Annexin V-FITC (BD Pharmingen, San Diego, CA, USA) and 5 μl of PI and incubated for 15 min at room temperature in the dark. Then, cytometry analysis was performed with CytoFLEX flow cytometry system and CytExpert (Beckman Coulter, USA).

### Western blotting

Protein extracted from cells were separated on SDS-PAGE and transferred to PVDF membranes (Millipore, Billerica, MA, USA). Membranes were blocked and then probed with antibodies against TopBP1 (ab2402, Abcam, 1/1000), ATR (2790S2, Cell Signaling Technology [CST], 1/1000), p-ATR (2853, CST, 1/1000) and CHK1 (2360, CST, 1/1000),p-CHK1 (2344s, CST, 1/1000) and GAPDH (1:1,000; Kangcheng Biology, Shanghai, China). The blots were washed by tris-buffered saline/Tween-20 solution and incubated with goat anti-rabbit or anti-mouse IgG (1:20,000; CST) at room temperature. The blots were visualized using Immobilon Western Chemiluminescent HRP Substrate (WBKLS0500, Merck Millipore, Germany).

### Statistical analysis

Statistical analysis was performed by using SPSS 22.0 software (SPSS Inc, IL, USA). Continuous variables were compared using the Student’s t-test for parametric data and the Mann-Whitney U test or Kruskal-Wallis test for non-parametric data. Categorical data were compared using the Chi-squared test and Fisher’s exact test. Kaplan-Meier plots were performed for survival analysis and the Cox proportional hazards regression model was conducted for univariate and multivariate survival analyses. Differences were considered statistically significant at p < 0.05 in all tests.

## References

[1] Ferlay J, Soerjomataram I, Dikshit R, Eser S, Mathers C, Rebelo M, Parkin DM, Forman D, Bray F. Cancer incidence and mortality worldwide: sources, methods and major patterns in GLOBOCAN 2012. Int J Cancer. 2015; 136:E359–86. 10.1002/ijc.29210 25220842

[2] Siegel RL, Miller KD, Jemal A. Cancer statistics, 2019. CA Cancer J Clin. 2019; 69:7–34. 10.3322/caac.21551 30620402

[3] Center MM, Jemal A, Lortet-Tieulent J, Ward E, Ferlay J, Brawley O, Bray F. International variation in prostate cancer incidence and mortality rates. Eur Urol. 2012; 61:1079–92. 10.1016/j.eururo.2012.02.054 22424666

[4] Mottet N, Bellmunt J, Bolla M, Briers E, Cumberbatch MG, De Santis M, Fossati N, Gross T, Henry AM, Joniau S, Lam TB, Mason MD, Matveev VB, et al. EAU-ESTRO-SIOG guidelines on prostate cancer. Part 1: screening, diagnosis, and local treatment with curative intent. Eur Urol. 2017; 71:618–29. 10.1016/j.eururo.2016.08.003 27568654

[5] Draisma G, Etzioni R, Tsodikov A, Mariotto A, Wever E, Gulati R, Feuer E, de Koning H. Lead time and overdiagnosis in prostate-specific antigen screening: importance of methods and context. J Natl Cancer Inst. 2009; 101:374–83. 10.1093/jnci/djp001 19276453 PMC2720697

[6] Karanika S, Karantanos T, Li L, Corn PG, Thompson TC. DNA damage response and prostate cancer: defects, regulation and therapeutic implications. Oncogene. 2015; 34:2815–22. 10.1038/onc.2014.238 25132269 PMC4333141

[7] Lord CJ, Ashworth A. The DNA damage response and cancer therapy. Nature. 2012; 481:287–94. 10.1038/nature10760 22258607

[8] Moudry P, Watanabe K, Wolanin KM, Bartkova J, Wassing IE, Watanabe S, Strauss R, Troelsgaard Pedersen R, Oestergaard VH, Lisby M, Andújar-Sánchez M, Maya-Mendoza A, Esashi F, et al. TOPBP1 regulates RAD51 phosphorylation and chromatin loading and determines PARP inhibitor sensitivity. J Cell Biol. 2016; 212:281–88. 10.1083/jcb.201507042 26811421 PMC4748576

[9] Yamane K, Kawabata M, Tsuruo T. A DNA-topoisomerase-II-binding protein with eight repeating regions similar to DNA-repair enzymes and to a cell-cycle regulator. Eur J Biochem. 1997; 250:794–99. 10.1111/j.1432-1033.1997.00794.x 9461304

[10] Wardlaw CP, Carr AM, Oliver AW. TopBP1: A BRCT-scaffold protein functioning in multiple cellular pathways. DNA Repair (Amst). 2014; 22:165–74. 10.1016/j.dnarep.2014.06.004 25087188

[11] Li L, Chang W, Yang G, Ren C, Park S, Karantanos T, Karanika S, Wang J, Yin J, Shah PK, Takahiro H, Dobashi M, Zhang W, et al. Targeting poly(ADP-ribose) polymerase and the c-myb-regulated DNA damage response pathway in castration-resistant prostate cancer. Sci Signal. 2014; 7:ra47. 10.1126/scisignal.2005070 24847116 PMC4135429

[12] d’Adda di Fagagna F. Living on a break: cellular senescence as a DNA-damage response. Nat Rev Cancer. 2008; 8:512–22. 10.1038/nrc2440 18574463

[13] Di Leonardo A, Linke SP, Clarkin K, Wahl GM. DNA damage triggers a prolonged p53-dependent G1 arrest and long-term induction of Cip1 in normal human fibroblasts. Genes Dev. 1994; 8:2540–51. 10.1101/gad.8.21.2540 7958916

[14] Wu Z, Li S, Tang X, Wang Y, Guo W, Cao G, Chen K, Zhang M, Guan M, Yang D. Copy number amplification of DNA damage repair pathways potentiates therapeutic resistance in cancer. Theranostics. 2020; 10:3939–51. 10.7150/thno.39341 32226530 PMC7086350

[15] Luo J, Luckenbaugh L, Hu H, Yan Z, Gao L, Hu J. Involvement of host ATR-CHK1 pathway in hepatitis B virus covalently closed circular DNA formation. mBio. 2020; 11:e03423–19. 10.1128/mBio.03423-19 32071277 PMC7029148

[16] Meyer F, Becker S, Classen S, Parplys AC, Mansour WY, Riepen B, Timm S, Ruebe C, Jasin M, Wikman H, Petersen C, Rothkamm K, Borgmann K. Prevention of DNA replication stress by CHK1 leads to chemoresistance despite a DNA repair defect in homologous recombination in breast cancer. Cells. 2020; 9:238. 10.3390/cells9010238 31963582 PMC7017274

[17] Bowen C, Ju JH, Lee JH, Paull TT, Gelmann EP. Functional activation of ATM by the prostate cancer suppressor NKX3.1. Cell Rep. 2013; 4:516–29. 10.1016/j.celrep.2013.06.039 23890999 PMC3838670

[18] Fokas E, Prevo R, Pollard JR, Reaper PM, Charlton PA, Cornelissen B, Vallis KA, Hammond EM, Olcina MM, Gillies McKenna W, Muschel RJ, Brunner TB. Targeting ATR in vivo using the novel inhibitor VE-822 results in selective sensitization of pancreatic tumors to radiation. Cell Death Dis. 2012; 3:e441. 10.1038/cddis.2012.181 23222511 PMC3542617

[19] Bartucci M, Svensson S, Romania P, Dattilo R, Patrizii M, Signore M, Navarra S, Lotti F, Biffoni M, Pilozzi E, Duranti E, Martinelli S, Rinaldo C, et al. Therapeutic targeting of Chk1 in NSCLC stem cells during chemotherapy. Cell Death Differ. 2012; 19:768–78. 10.1038/cdd.2011.170 22117197 PMC3321626

[20] Taricani L, Wang TS. Rad4TopBP1, a scaffold protein, plays separate roles in DNA damage and replication checkpoints and DNA replication. Mol Biol Cell. 2006; 17:3456–68. 10.1091/mbc.e06-01-0056 16723501 PMC1525248

[21] Kumagai A, Lee J, Yoo HY, Dunphy WG. TopBP1 activates the ATR-ATRIP complex. Cell. 2006; 124:943–55. 10.1016/j.cell.2005.12.041 16530042

[22] Lindsey-Boltz LA, Sancar A. Tethering DNA damage checkpoint mediator proteins topoisomerase IIbeta-binding protein 1 (TopBP1) and claspin to DNA activates ataxia-telangiectasia mutated and RAD3-related (ATR) phosphorylation of checkpoint kinase 1 (Chk1). J Biol Chem. 2011; 286:19229–36. 10.1074/jbc.M111.237958 21502314 PMC3103301

[23] Karanika S, Karantanos T, Li L, Wang J, Park S, Yang G, Zuo X, Song JH, Maity SN, Manyam GC, Broom B, Aparicio AM, Gallick GE, et al. Targeting DNA damage response in prostate cancer by inhibiting androgen receptor-CDC6-ATR-Chk1 signaling. Cell Rep. 2017; 18:1970–81. 10.1016/j.celrep.2017.01.072 28228262 PMC5349188

[24] Schmidt U, Wollmann Y, Franke C, Grosse F, Saluz HP, Hänel F. Characterization of the interaction between the human DNA topoisomerase IIbeta-binding protein 1 (TopBP1) and the cell division cycle 45 (Cdc45) protein. Biochem J. 2008; 409:169–77. 10.1042/BJ20070872 17887956

[25] Gerhardt J, Guler GD, Fanning E. Human DNA helicase B interacts with the replication initiation protein Cdc45 and facilitates Cdc45 binding onto chromatin. Exp Cell Res. 2015; 334:283–93. 10.1016/j.yexcr.2015.04.014 25933514 PMC4439256

[26] Liu T, Lin YH, Leng W, Jung SY, Zhang H, Deng M, Evans D, Li Y, Luo K, Qin B, Qin J, Yuan J, Lou Z. A divergent role of the SIRT1-TopBP1 axis in regulating metabolic checkpoint and DNA damage checkpoint. Mol Cell. 2014; 56:681–95. 10.1016/j.molcel.2014.10.007 25454945 PMC4386886

[27] Mooser C, Symeonidou IE, Leimbacher PA, Ribeiro A, Shorrocks AK, Jungmichel S, Larsen SC, Knechtle K, Jasrotia A, Zurbriggen D, Jeanrenaud A, Leikauf C, Fink D, et al. Treacle controls the nucleolar response to rDNA breaks via TOPBP1 recruitment and ATR activation. Nat Commun. 2020; 11:123. 10.1038/s41467-019-13981-x 31913317 PMC6949271

[28] Liu K, Bellam N, Lin HY, Wang B, Stockard CR, Grizzle WE, Lin WC. Regulation of p53 by TopBP1: a potential mechanism for p53 inactivation in cancer. Mol Cell Biol. 2009; 29:2673–93. 10.1128/MCB.01140-08 19289498 PMC2682038

[29] Seol HJ, Yoo HY, Jin J, Joo KM, Kim HS, Yoon SJ, Choi SH, Kim Y, Pyo HR, Lim DH, Kim W, Um HD, Kim JH, et al. The expression of DNA damage checkpoint proteins and prognostic implication in metastatic brain tumors. Oncol Res. 2011; 19:381–90. 10.3727/096504011x13123323849654 22329197

[30] Wang C, Liu Q, Huang M, Zhou Q, Zhang X, Zhang J, Xie R, Yu Y, Chen S, Fan J, Chen X. Loss of GATA6 expression promotes lymphatic metastasis in bladder cancer. FASEB J. 2020; 34:5754–66. 10.1096/fj.201903176R 32103545

[31] Gu P, Chen X, Xie R, Xie W, Huang L, Dong W, Han J, Liu X, Shen J, Huang J, Lin T. A novel AR translational regulator lncRNA LBCS inhibits castration resistance of prostate cancer. Mol Cancer. 2019; 18:109. 10.1186/s12943-019-1037-8 31221168 PMC6585145

